# The Functional Roles of the Src Homology 2 Domain-Containing Inositol 5-Phosphatases SHIP1 and SHIP2 in the Pathogenesis of Human Diseases

**DOI:** 10.3390/ijms25105254

**Published:** 2024-05-11

**Authors:** Spike Murphy Müller, Manfred Jücker

**Affiliations:** Institute of Biochemistry and Signal Transduction, University Medical Center Hamburg-Eppendorf, 20246 Hamburg, Germany; spike.mueller@stud.uke.uni-hamburg.de

**Keywords:** SHIP1, SHIP2, PI3K/AKT signaling pathway, phosphoinositide signaling, intracellular signaling, human diseases, hematopoietic cancer, cancer, autoimmune diseases, therapeutic targets

## Abstract

The src homology 2 domain-containing inositol 5-phosphatases SHIP1 and SHIP2 are two proteins involved in intracellular signaling pathways and have been linked to the pathogenesis of several diseases. Both protein paralogs are well known for their involvement in the formation of various kinds of cancer. SHIP1, which is expressed predominantly in hematopoietic cells, has been implicated as a tumor suppressor in leukemogenesis especially in myeloid leukemia, whereas SHIP2, which is expressed ubiquitously, has been implicated as an oncogene in a wider variety of cancer types and is suggested to be involved in the process of metastasis of carcinoma cells. However, there are numerous other diseases, such as inflammatory diseases as well as allergic responses, Alzheimer’s disease, and stroke, in which SHIP1 can play a role. Moreover, SHIP2 overexpression was shown to correlate with opsismodysplasia and Alzheimer’s disease, as well as metabolic diseases. The SHIP1-inhibitor 3-α-aminocholestane (3AC), and SHIP1-activators, such as AQX-435 and AQX-1125, and SHIP2-inhibitors, such as K161 and AS1949490, have been developed and partly tested in clinical trials, which indicates the importance of the SHIP-paralogs as possible targets in the therapy of those diseases. The aim of this article is to provide an overview of the current knowledge about the involvement of SHIP proteins in the pathogenesis of cancer and other human diseases and to create awareness that SHIP1 and SHIP2 are more than just tumor suppressors and oncogenes.

## 1. Introduction

The goal of this review is to summarize the current knowledge concerning the diseases that are potentially caused or promoted by either the up- or downregulation of the src homology 2 (SH2) domain containing inositol 5-phosphatases SHIP1 and SHIP2 or their involvement in the pathogenesis in any other way. The two SHIP paralogs are known tumor suppressors and can, in some cases, function as oncogenes as well. Further, the involvement in inflammatory diseases and allergic responses, as well as Alzheimer’s disease, metabolic diseases, opsismodysplasia, vascular diseases, and stroke, has been indicated in the literature. At first, the current state of knowledge about the structure and intracellular signaling of the SHIP proteins is described, followed by their involvement in human diseases. Therapeutic methods, which are under investigation for clinical usage, are discussed as well. Most of the clinical research is focused on pharmacological substances such as inhibitors or activators that affect the catalytic activity of SHIP, which may be used in the treatment of leukemia, type 2 diabetes, and Alzheimer’s disease, as well as in patients with cystitis-induced bladder pain.

## 2. The Structure and Function of SH2 Domain-Containing Inositol Phosphatases

### 2.1. General Structure of SH2 Domain-Containing Inositol Phosphatases

**Figure 1:**
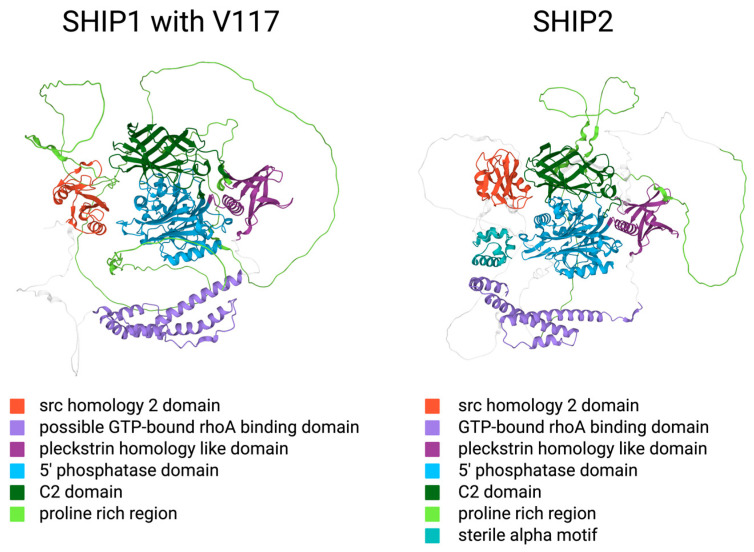
AlphaFold protein structure predictions of the consensus sequences of SHIP1, with Va-lin117, and SHIP2 [7,8] (created with BioRender.com). In this figure, the order of the domains from top to bottom is equivalent to the order of the domains within the corresponding protein from carboxy-terminus to amino-terminus.

The SHIP proteins consist of multiple domains (Figure 1). Both SHIP1 and SHIP2 contain an amino-terminal SH2 domain as well as a catalytic 5′-phosphatase (5-PPase) domain, which is enclosed by a pleckstrin homology-like (PHL) domain, that is located facing toward the amino terminus, and a C2 domain, which lies further in the direction of the carboxy terminus. The c-terminal region consists of PXXP and NPXY motifs [1]. Those PXXP and NPXY motifs are located in a proline-rich region (PRR) [2].

### 2.2. Structural Similarities and Differences between the SHIP Paralogs

The amino acid sequences of SHIP1 and SHIP2 are reported to be fairly similar [3], [1]. A sequence alignment with SnapGene shows 57.4% identity in the nucleotide sequence. Furthermore, several secondary structures and domains are located in the same regions within the two proteins (Figure 1). However, the enzymes are expressed in different tissues, and even though the 5-Ppase domain specifically seems to be very similar, there are regions with less sequence homology. 

SHIP1, also known as p145-SHIP due to its molecular mass of 145 kD, is encoded within the *INPP5D* gene on chromosome 2. The SHIP1 protein consists of approximately 1190 amino acids [4]. Since the publication of those data, the scientific community has agreed upon two single amino acid polymorphisms as consensus sequences, which can be found, for example, in the NCBI database or in UniProt. The Asian isoform consists of 1189 amino acids, whereas the Caucasian isoform lacks the Valin117 and therefore contains 1188 amino acids. The protein contains an amino-terminal SH2 domain [4]. SH2 domains are commonly known as loci for protein–protein interactions [4]. Specifically, SH2 domains are able to bind phosphorylated tyrosine residues with high affinity in one of their binding pockets, while the second pocket interacts with the amino acid, which lies three positions downward. Further, the SHIP1 sequence includes two NPXY motifs in the carboxy-terminal region, which, after the phosphorylation of tyrosine residues, potentially interact with phosphotyrosine-binding (PTB) domains of other proteins [4]. In the Caucasian polymorphism with Val117, the two NPXY motifs lie within the residues 912–915 and 1019–1022. In uniport, three src homology 3 (SH3) binding motifs and a disabled-2 (DAB2) binding motif are recorded within the structure of the SHIP1 protein [5]. Those consist of a PELPPR motif in the linker between the SH2 domain and the PHL domain, a PISPKK motif, and a PKMPRKEPPPCP motif in the PRR, and the DAB2 interaction motif is reported between those last mentioned motifs in residues 1016 to 1030 [5]. In addition, the existence of five nuclear export signals (NESs) and two nuclear localization signals (NLSs) was reported [6]. PXXP motifs are also found in the c-terminal region and in the linker between the SH2 and PHL domains, which could serve as binding sites for SH3 domain-containing proteins [4]. The catalytic domain consists of around 400 to 500 amino acids and lies in the center of the protein [4]. SHIP1 also contains a C2 domain, which is localized in residues 725 to 863 [3].

Thomas et al. described the sequence and structure of SHIP2 in the following way in 2017. The gene for SHIP2 is called *INPPL1* and is located on chromosome 11. After the transcription, the pre-mRNA undergoes the mechanism of alternative splicing, which generates two currently known isoenzymes with 1258 (isoform 1) and 1016 (isoform 2) amino acids. The second isoenzyme is missing the first 242 amino acids. The protein includes two PXXP motifs in residues 139–142 and 140–144, which can bind SH3 domains, and several other PXXP domains that could have similar functions. Another range of residues near the carboxy terminus also represents an SH3 domain binding motif (PPAPPR motif). Further, there is an NPXY motif consisting of residues 983–986 in which there exists a tyrosine residue (Y986) that represents a phosphorylation site. NPXY motifs commonly interact with phosphotyrosine-binding domains. Residues 742 to 884 seem to be similar to the corresponding C2 domain in SHIP1, which leads to the assumption that this part may constitute a C2 domain as well. The c-terminus of SHIP2 consists of a ubiquitin-interacting motif (UIM) and a following sterile alpha motif (SAM) domain. Both the UIM and the SAM domains are not found in SHIP1 [3].

### 2.3. The Intracellular Function of SHIP1 and SHIP2

**Figure 2:**
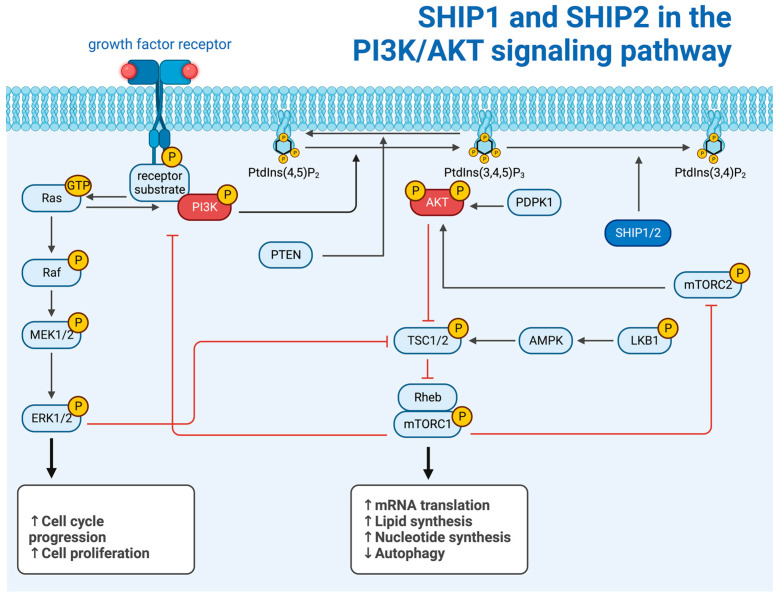
SHIP1 and SHIP2 in the PI3K/AKT signaling pathway (created with BioRender.com).

In physiological conditions, SHIP1 is predominantly expressed in hematopoietic cells, including but not limited to erythrocytes, macrophages, and granulocytes [9]. SHIP1 is essential for the regulation of survival, proliferation, and function of those cells, especially in cells functioning in the immune system [9]. In addition, the expression of SHIP1 in healthy osteoblasts has been shown [10]. SHIP2, however, is expressed ubiquitously [11]. The expression of SHIP1 is regulated by extracellular signals from cytokines and chemokines, among others [12]. Both SHIP paralogs function primarily as regulators of the membrane-bound phosphoinositides as well as soluble inositol phosphates and thereby have an impact on various intracellular signaling pathways [1] (Figure 2). Those phosphoinositides are anchored in the plasma membrane with their acyl chains, which constitute diacylglycerol (DAG) and are linked to the C1 atom of the inositol ring [1]. There are eight different phosphoinositides, which mainly differ in the amount and positioning of phosphate groups, which can interact with free hydroxyl groups in the inositol ring under the formation of phosphate ester bonds [1]. With its six carbon atoms, a multitude of phosphoinositides could be formed; however, only a limited number of eight variants are actually being produced [1]. Their localization on the inner surface of the cell membrane makes them capable of transferring information from transmembrane receptors and thus activating intracellular signaling pathways in response to changes in the extracellular milieu [1]. SHIP1 is known to regulate intracellular phosphoinositide levels by converting phosphatidylinositol-3,4,5-trisphosphate (PtdIns(3,4,5)P_3_) into phosphatidylinositol-3,4-bisphosphate (PtdIns(3,4)P_2_) and thereby antagonizing the activity of phosphatidylinositol-3,4,5-trisphosphate 3-kinase (PI3K) [13]. SHIP1 can also dephosphorylate Inositol-1,3,4,5-tetrakisphosphate (Ins(1,3,4,5)P_4_), which is lacking the DAG and therefore is not directly associated with the cellular membrane, thereby creating Inositol-1,3,4-trisphosphate (Ins(1,3,4)P_3_) [14]. SHIP1 is limited to those phosphoinositides as substrates because it requires a phosphate group on the C3 atom in order to successfully bind the substrate [14]. SHIP2 has been shown to dephosphorylate phosphatidylinositol-4,5-bisphosphate (PtdIns(4,5)P_2_), as well as phosphatidylinositol-3,5-bisphosphate (PtdIns(3,5)P_2_) and PtdIns(3,4,5)P_3_, all on the C5 atom [15]. SHIP2 binds PtdIns(3,5)P_2_ and PtdIns(3,4,5)P_3_ with approximately the same affinity [15]. The probably most important and most researched signaling pathway that is heavily regulated by PtdIns and in which the SHIP proteins are shown to play an important role in regulating just those phosphoinositides and consequently the signaling chain is the PI3K/AKT pathway [1] (Figure 2).

This pathway starts with the binding of an extracellular ligand to receptor tyrosine kinases (RTKs). Two subunits form homodimers. Those subunits possess intrinsic tyrosine kinase activity. They phosphorylate one another on tyrosine residues. Those residues now represent binding sites for the PI3K via adapter proteins such as the insulin receptor substrate (IRS). The PI3K then phosphorylates and thereby converts PtdIns(4,5)P_2_ into PtdIns(3,4,5)P_3_. PtdIns(3,4,5)P_3_ is directly associated with the inner surface of the plasma membrane and binds the protein kinase B (PKB = AKT). The 3-phosphoinositide-dependent protein kinase 1 (PDPK1) is also recruited to the membrane by PtdIns(3,4,5)P_3_. The proximity of PDPK1 to AKT then enables PDPK1 to phosphorylate and activate AKT (Figure 2). Seeing that the PI3K converts PtdIns(4,5)P_2_ to PtdIns(3,4,5)P_3_ by attaching a phosphate to C3 and SHIP1 converts PtdIns(3,4,5)P_3_ to PtdIns(3,4)P_2_ by removing a phosphate at the C5 atom of the inositol ring, SHIP1 is often regarded as an antagonist to the PI3K. However, it is more complex than this.

First, PtdIns(3,4)P_2_ as well as PtdIns(3,4,5)P_3_ have been shown to interact with AKT and activate the PI3K/AKT signaling pathway under certain circumstances [14]. Furthermore, the SHIP1 substrate PtdIns(3,4,5)P_3_ is necessary for the activation of Tec family kinases, which then activate the phospholipase C gamma (PLC-γ) [14]. The activation of PLC-γ in turn is necessary for the generation of Inositol-1,4,5-trisphosphate (Ins(1,4,5)P_3_), which is further phosphorylated by the Inositol-1,4,5-trisphosphate 3-kinase (IP3K) to Inositol-1,3,4,5-tetrakisphosphate (Ins(1,3,4,5)P_4_) [14]. This means the reduction in the PtdIns(3,4,5)P_3_ content by SHIP1 can lead to a reduced concentration of Ins(1,4,5)P_3_ and Ins(1,3,4,5)P_4_.

But SHIP1 is not in all cases reliant on its enzymatic activity in order to influence intracellular signaling. In addition, SHIP1 competitively binds DNAX-activation protein 12 (DAP12), inhibiting the spleen tyrosine kinase (Syk) from binding [16], and both SHIP paralogs can also display receptor masking functions, where they bind to the receptor with their SH2 domain and thereby cover the binding sites of other enzymes [10]. The SHIP proteins can also bind other proteins and enzymes, which is facilitated by protein–protein interactions between the carboxy-terminal proline-rich regions and NPXY motifs of SHIP with SH3 domains or PTB motifs of the other proteins, respectively [10].

## 3. The Role of SHIP1 in Human Diseases

**Figure 3:**
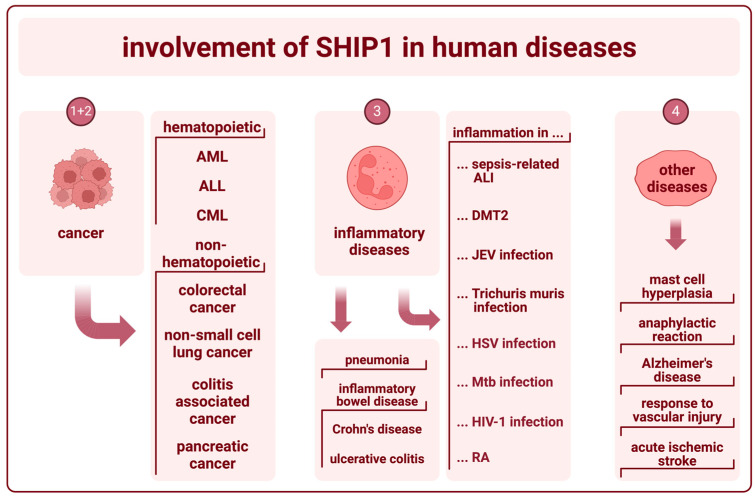
Involvement of SHIP1 in human diseases (created with BioRender). The numbers herein refer to the paragraphs with the corresponding subheading numbers under “3. The role of SHIP1 in human diseases”, wherein the involvement of SHIP1 in the pathogenesis of those diseases is further elucidated.

The primary pathological role of SHIP1 lies in the development and regulation of hematopoietic cancers and inflammatory bowel diseases (IBD). However, SHIP1 expression was shown in other sorts of cancerous cells, seems to be a relevant factor in the formation and regulation of other inflammatory diseases, as well as in Alzheimer’s disease, and is believed to play a role in primary hemostasis (Figure 3).

### 3.1. SHIP1 in Hematopoietic Cancer

First, we will have a look at the effects of SHIP1 on hematopoietic cancers. This area seems to be best researched regarding the pathological mechanisms of SHIP1 in humans.

In general, SHIP1 and SHIP2 both catalyze the dephosphorylation of PtdIns(3,4,5)P_3_ to PtdIns(3,4)P_2_. PtdIns(3,4,5)P_3_ is well known for its oncogenic potential resulting from recruiting AKT and inducing the phosphorylation of Thr308 through PDPK1, thereby triggering cancer cell survival [1]. AKT is further phosphorylated at Ser473 by the mammalian target of rapamycin complex 2 (mTORC2), which in turn activates AKT to a higher degree than only the phosphorylation on Thr308 [1]. Reduced expression of the *INPP5D* gene encoding SHIP1 is seen in several human leukemias, like acute myeloid leukemia (AML), acute lymphoblastic leukemia (ALL), and chronic myeloid leukemia (CML) [15]. Upregulation of the PI3K/AKT signaling pathway is reported to be present iIn up to 70% of all patients with AML [17]. The relevance of SHIP1 in this context was shown via lentiviral transfection of CD34-positive AML cells with *INPP5D* and verification of reduced proliferation [17]. Furthermore, the mutation V684E was found in AML, which lies in the phosphatase domain of SHIP1 and was shown to reduce the catalytic effect of the protein, which in turn raises AKT activity [15]. The possible role of SHIP1 as a tumor suppressor in T-ALL cells is suggested on the basis of a strong downregulation of SHIP1 in T-ALL cells with upregulated PI3K/AKT signaling [18]. In CML, the BCR/ABL fusion transcript reduces the expression of SHIP1, which indicates a role for SHIP1 as a tumor suppressor in CML [19].

Regarding B cells, SHIP1^−/−^ mice are often osteoporotic and show a variety of diseases like myeloid cell infiltration and inflammation of the lung, a shortened lifespan, and spontaneous B cell lymphoma [15]. There seem to be strong suggestions that, at least in mice, SHIP1 is involved in B cell proliferation and therefore regulates the amount of active B cells in the peripheral blood [15]. However, higher amounts of post-transcriptional regulation of the SHIP1-Gene *INPP5D* with the micro-RNA miR-155 have been shown to negatively affect the prognosis of B cell lymphoma in humans [1]. In one more study, it is stated that a loss of function or deficiency of SHIP2 in Burkitt lymphoma cells does not affect pro-survival PI3K-AKT signaling. Reduced function of both SHIP paralogs, however, is believed to lead to increased susceptibility to inhibition of the PI3K-AKT-signaling pathway as a possible target in treatment [20].

### 3.2. SHIP1 in Other Types of Cancer

Even though SHIP1 is mostly known for its involvement in the occurrence of hematopoietic cancer, which seems logical when considering that SHIP1 is mostly expressed in hematopoietic cells, the expression of SHIP1 is also described in various other types of cancer.

SHIP1 was shown to be upregulated in colorectal cancer [21]. A correlation between patient survival and SHIP1 expression could not be found [21]. Rather, SHIP1 expression was found to be inversely correlated with tumor grade, vascular invasion, and lymph node metastasis [21]. It was also shown that SHIP1 is expressed in non-small cell lung cancer (NSCLC) [22]. Furthermore, a reduced SHIP1 expression is correlated with reduced survival rates in NSCLC patients [22]. Further, the role of SHIP1 in the development of colitis-associated cancer (CAC) is less understood in the literature. It is known that SHIP1 and other negative regulators suppress the inflammatory response induced by toll-like receptor 4 (TLR4) signaling when the ligand, a lipopolysaccharide (LPS), is bound [23]. Additionally, LPS stimulation was shown to increase the transcription of miR-155. The miRNA is a negative regulator of the expression of SHIP1. The authors believe that the interaction between the toll-like receptor 4 (TLR4) and miR-155 could play a role in the facilitation of the disease. Theoretically, TLR4 activation could induce miR-155 expression, which downregulates SHIP1 expression, which in turn could lead to reduced suppression of the inflammatory response. If SHIP1 could be a significant player in this interaction and in the development of CAC is currently unknown [23]. SHIP1 also was reported to be relevant in pancreatic cancer (PC). One study states that PC can induce miR-155 and thereby reduce SHIP1 expression, which positively impacts antitumor immunity in mice [12]. However, this study focuses on SHIP activity manipulation as a possible therapy, so this will be further discussed in the next chapter.

### 3.3. SHIP1 in Inflammatory Diseases

Multiple studies show that SHIP1 deficiencies can cause lethal pneumonia and inflammation in the ileum in mice, with a strong resemblance to human Crohn’s disease (CD). This was often shown via double knockout mice (SHIP1^−/−^ mice). The most prevalent explanation for the lung inflammation, according to Fernandes et al. in 2013, appears to be a myeloproliferative process, with a reduction in the alveolar space brought on by a reduced turnover in the SHIP1-deficient myeloid cells. The inflammation of the ileum is believed to be caused by dysregulation of the T cell homeostasis and myeloid infiltration, particularly with neutrophil granulocytes [10]. SHIP1 is known to also have a relevant role in the homeostasis of CD4+ T cells in the blood. Fernandes et al. stated in 2018 that around 15% of patients with inflammatory bowel disease (IBD) are shown to have a severe lack of SHIP1 protein in their blood samples and reduced numbers of CD4+ T cells in circulation. In the study I am referring to, the patient pool consisted of individuals with Crohn’s disease (CD) and ulcerative colitis (UC). That being said, a SHIP1 deficiency alone is not known to be causal for the emergence of those diseases, but it appears to be a factor in the increased severity that CD displays [24]. Additionally, it is known that lipopolysaccharide (LPS)-responsive beige-like anchor (LRBA) deficiencies play a role in the development of inflammatory bowel disease (IBD) [25]. This study examined the development of lethal colitis if the mice in the animal model consumed dextran sulfate sodium (DSS). Furthermore, they showed that increased activity of SHIP1 partially mitigated the development of lethal colitis in this context and concluded that SHIP1 agonization could prove to be an effective protective measure in LRBA deficiencies [25]. Another study looked at the development of lung inflammation in sepsis-related acute lung injury (ALI) and the role of serum exosomes in the pathogenesis. They could show that the overexpression of miR-155 in macrophages leads to strongly depressed intracellular concentration of SHIP1 in mice [26]. The authors concluded that miR-155 targets the 3′-UTR of the SHIP1-mRNA and thereby reduces SHIP1 translation, which in turn leads to lower levels of SHIP1. With respect to the serum exosomes, they stated that exosome-derived miR-155 also suppresses SHIP1 in macrophages. In this case, the study could show that the reduced expression of SHIP1 leads to increased cellular proliferation and inflammation in macrophages [26]. One other study by Berry et al. suggests that interleukin 10 (IL-10) hypo-responsiveness could play a role in chronic inflammation, which occurs in diabetes mellitus type 2 (DMT2). In this study from 2016, they activated SHIP1 in macrophages, which were cultured under high-glucose conditions. The activation of SHIP1 was shown to reverse the IL-10 hypo-responsiveness, suggesting that SHIP1 also plays a role in the signaling of chronic inflammation in DMT2. For the pathway, they found that SHIP1 activity is required for IL10 activity. IL10 then suppresses the translation of the tumor necrosis factor alpha (TNF-α) mRNA. The authors further discussed that SHIP1 agonists could potentially be used in the therapy of inflammation induced by metabolic conditions [27]. Moreover, SHIP1 could play a role in the neuroinflammatory processes involved in the pathogenesis of the Japanese encephalitis virus (JEV) [28]. The JEV-induced micro-RNA miR-155 downregulates SHIP1 by post-transcriptional regulation, thereby positively modifying the activity of NF-κB and the immune response in mouse models. In contrast, it could be shown that increased amounts of miR-155 in human microglial cells lead to the dampening of the immune response through other pathways [28]. The actual role of SHIP1 in this context is not sufficiently analyzed to give a clear answer as to whether SHIP1 plays a significant role in the pathogenicity and cell damage in JEV-infected patients [28]. However, there are better data concerning the role of SHIP1 in the infection caused by the intestinal parasitic worm *Trichuris muris.* One study by Hadidi et al. in particular could identify that SHIP1 could play a potentially important part in the pathogenicity of the infection, which presents with the accumulation of macrophages. Because of the well-determined role of SHIP1 in the macrophages’ proliferation signaling, the authors used mouse models with a myeloid cell-specific deletion of SHIP1 to determine the specific function of SHIP1. They could show that the macrophages in mice with the SHIP1 deletion produced higher amounts of interleukin 12 (IL-12), which led to a non-protective T_H_1 cell response [29]. One other study could show anti-inflammatory properties of SHIP1 regarding herpes simplex keratitis (HSK) which is induced by an infection of the cornea with the herpes simplex virus. In the study, a SHIP1 activator was injected subconjunctivally, which led to reduced inflammation, faster recovery, and less CD4+ T cell infiltration [30]. Another study looked at the host–pathogen interaction in patients infected with Mycobacterium tuberculosis (Mtb). They report that miR-155 has two opposing roles in this interaction [31]. One of them is the promotion of the survival of infected macrophages, and the other one is the promotion of the survival of Mtb-recognizing T cells. The authors suggest that the miRNA mediates this through the SHIP1/AKT pathway [31]. Furthermore, there is a study that investigated the role of SHIP1 in the loss of the typical effector function of CD3-CD56-CD16^+^ natural killer cells in human immunodeficiency virus 1 (HIV-1) infections. They could show a correlation between the impairment of the cells and the reduced production of perforin, as well as an increased production of SHIP1 [32]. The study did not further differentiate between those two variables. Whether the downregulation of perforin or the upregulation of SHIP1 is the dominant trigger for the loss of important functions or if it is based on the interplay of both cannot be determined with the information from this study. Another study reported the upregulation of miR-155 and consequent downregulation of SHIP1 in macrophages in the synovial membrane and synovial fluid in patients with rheumatoid arthritis (RA) [33].

### 3.4. SHIP1 in Other Diseases

First, it was shown that the loss of SHIP1, primarily in mast cells, plays a role in mast cell hyperplasia and heightened anaphylactic response in double knockout (SHIP1^−/−^) mice [34]. However, the role of SHIP1 in humans concerning the pathophysiology of allergies and allergic responses has not been elucidated so far. Second, there was evidence reported recently that SHIP1 could take part in the development of Alzheimer’s disease. It has been reported that SHIP1 in brain microglia cells could be significantly involved in the onset and development of late-onset Alzheimer’s disease (LOAD) [35]. Specifically, it was found that Alzheimer’s disease (AD) is correlated with an increase in the expression of *INPP5D*, and the elevated SHIP1 levels are believed to be associated with microglial markers and amyloid plaque density. Furthermore, a correlation was reported between the amount of upregulation of *INPP5D* expression and the progression of AD [35]. Third, one study from 2007 could show that SHIP1 also seems to be relevant in the activation of thrombocytes. They found that SHIP^−/−^ mice had a worse response to vascular injury, which consisted of less thrombocyte aggregation and fibrinogen binding as well as longer tail bleeding times. Although this study focused on the physiological function of SHIP1 in blood platelets, this could be an indication that a loss of function or the loss of SHIP1 could negatively affect primary hemostasis [36]. And last, there is a study that investigated blood protein levels of SHIP1 in Chinese patients with acute ischemic stroke (AIS). They found that SHIP1 protein blood levels of ≥ 1.550 pg/ml could be a marker for the risk of AIS development in the Chinese population [37]. However, the study did not further examine the role of SHIP1 in the development of the disease.

## 4. SHIP1 Modulation as a Possible Treatment in Human Diseases

**Figure 4:**
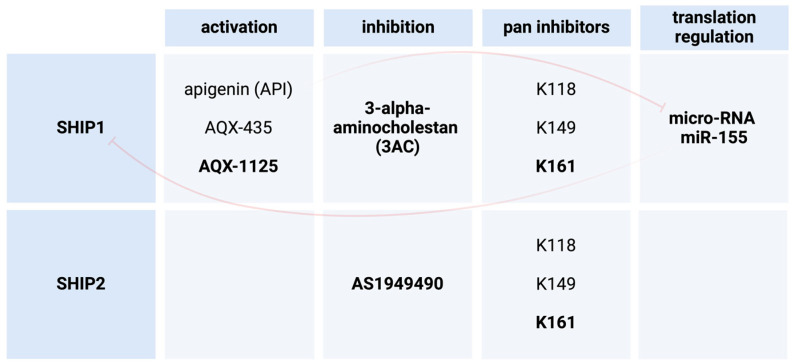
Pharmacological SHIP paralog regulators (created with BioRender). API regulates the function of SHIP1 via inhibition of miR-155. AQX-1125 has been tested in clinical studies.

The use of pharmacological agents for the activation or inhibition of SHIP1 has been described for different human diseases (Figure 4). One study demonstrates that the inhibition of SHIP1 in SHIP1-expressing AML cells in vitro decreases cell growth and survival by promoting apoptosis [38]. In this study, they used the inhibitor 3-alpha-aminocholestan (3AC) on the cell line KG-1. They concluded that SHIP1 inhibitors could potentially be used in the treatment of different leukemias, like acute myeloid leukemia (AML) [38]. One other study investigated the usage of SHIP1 modulators for therapeutic use in pancreatic cancer. They were able to show that increased expression of SHIP1 led to a regression in pancreatic tumors in mice [12]. In this study, they investigated the bioflavonoid apigenin (API) and its SHIP1 activating effects. The elevated expression of SHIP1 is believed by the authors to probably be due to the inhibition of micro-RNA miR-155 whose expression is induced by pancreatic cancer. miR-155 is a known negative regulator for SHIP1 translation [12]. There are studies suggesting that the pharmacological activation of SHIP1, for instance via AQX-435, could be a possible treatment in B cell neoplasms. They specifically showed that the reduction in AKT signaling leads to the induction of apoptosis in vitro and reduced lymphoma growth in mouse models [39]. Further, the data of one more experimental study demonstrated the induction of cell death in chronic lymphocytic leukemia (CLL) through the inhibition of SHIP1 [40]. The cell death of the CLL cells seems to be triggered by reactive oxygen species (ROS) [40]. This ROS-mediated cell death was observed as a result of heightened AKT activity and subsequently increased mitochondrial respiration, which leads to the accumulation of ROS [40]. Additionally, the small molecule activator AQX-1125 was shown to potentially be useful in the future treatment of interstitial cystitis [41].

### 4.1. 3AC as a SHIP1 Inhibitor in Leukemia

Brooks et al. first identified 3-α-aminocholestan (3AC) as an inhibitor of the catalytic activity of SHIP1 by using a fluorescent polarization assay and then treated mice with this SHIP1 inhibitor. They treated adult mice for seven days with the compound and compared the quantity of myeloid-derived immune cells in the secondary lymphoid tissue with that in normal and vehicle-injected mice. They report increased amounts of myeloid-derived immune cells after treatment with 3AC. Furthermore, they postulated and confirmed that the expansion of the myeloid-derived immune cells in the periphery should reduce the priming of T cells in response to allogeneic cells. Next, they examined the granulocyte levels in mice treated with 3AC and found drastically increased levels of circulating neutrophil granulocytes, which corresponded to their hypothesis. They further looked into the possibility that SHIP1 inhibition might be able to rescue the production of essential blood cells in myelosuppressed mice. After irradiating mice and thereby creating this decreased hematopoietic activity of the damaged bone marrow, they again treated the mice over the course of seven days with 3AC and found significantly increased red blood cell counts, which did not drop below normal in comparison to mice treated with the vehicle or mice with no intervention. Finally, there were concerns in the work group that the inhibition of SHIP1 could lead to enhanced growth and survival in leukemia cells. They treated SHIP1-expressing AML cells (KG-1) with their inhibitor and were able to detect a restriction of growth and survival. They concluded that 3AC treatment reduces intracellular PtdIns(3,4)P_2_ levels and thereby leads to a reduction in growth and survival in SHIP1-expressing hematopoietic cancer cells [38].

### 4.2. 3AC as a SHIP1 Inhibitor in Leishmania Major and Leishmania Donovani

A recent study by Chowdhury et al. from 2023 tested the formerly mentioned 3-α-aminocholestan with respect to the effects in mice with Leishmaniasis, a parasitic disease. In humans, the *Leishmania major* parasite, which is known to be causal for cutaneous Leishmaniasis, is commonly transmitted in west and middle Asia by sandflies of the genus *Phlebotomus* but also occurs in North and South America as well as in Europe [42]. The *Leishmania donovani* parasite causes visceral Leishmaniasis [43]. Chowdhury et al. found that 3AC administration affected the production of TNF-α, IL-12, and interferon gamma (IFN-γ) positively, which the authors called anti-leishmanial cytokines, and reduced the production of IL-10 and TGF-β, called pro-leishmanial cytokines by the authors, as well as activated NO synthesis. Those effects were allocated to the inhibition of SHIP1; however, the authors did not show any causal link between SHIP1 inhibition and the resulting effects directly. Three plausible pathways were mentioned, which could be investigated further. Those include changes in transcription factor activation, reduced survival of the amastigote stage of the parasite due to higher PtdIns(3,4,5)P_3_ contents, and reduced relocation of SHIP1 to subcellular target locations, where a higher SHIP1 concentration would act in a function beneficial to the leishmania parasite [44].

### 4.3. SHIP1 Activation through miRNA Inhibition

Husain et al. conducted a study in 2022 to identify apigenin (API) as a miR-155 inhibitor. They previously found that apigenin could increase the expression of SHIP1 in pancreatic cancer, which leads to the conversion of monocytic myeloid-derived suppressor cells into pro-inflammatory tumor-associated macrophages with an M1 phenotype. This conversion was accompanied by tumor regression. With this study, they wanted to uncover the pathway. First, they treated different pancreatic cancer cell lines from mice and humans with API or the vehicle (control). Afterward, they used reverse transcription-quantitative polymerase chain reaction (RT-qPCR) to verify that apigenin indeed suppressed the expression of miR-155 in pancreatic cancer cells. They found that miR-155 was significantly less expressed in API-treated cells in contrast to a control group that was treated with only the vehicle. Furthermore, it was shown that the concentration of apigenin was correlated with the induction of apoptosis. Next, they transfected different pancreatic cell lines with a known miR-155 inhibitor or scrambled miRNA (control) and found less miR-155 expression and reduced viability in comparison to the control group. Hereby, they could show that treatment with apigenin leads to a reduction in miR-155 expression and consequently acts in an anti-carcinogenic manner. After that, the work group wanted to further investigate if the combination of a miR-155 inhibitor with apigenin would have a synergistic effect on the survival of pancreatic cancer cells. They again transfected a cell line with the known miR-155 inhibitor or scrambled miRNA (control) and treated both with apigenin. They found that the miR-155 expression was lower in the cell line that was transfected with the miR-155 inhibitor, indicating a synergistic intracellular action. The reduction in miR-155 expression was evermore accompanied by an increase in both the transcription of the *INPP5D* gene and the translation of the SHIP1 protein [12].

### 4.4. AQX-435 as a SHIP1 Activator in B Cell Neoplasms

Lemm et al. reported that the SHIP1 activator AQX-435 could potentially be useful for the therapy of B-cell neoplasms characterized by the activation of PI3K signaling. They first investigated the effect of AQX-435 on AKT phosphorylation as a response to anti-IgM stimulation in CLL cells. They found that the pharmacological agent reduced the phosphorylation of AKT in a dose-dependent manner. Furthermore, they showed that the decrease in PtdIns(3,4,5)P_3_ is the relevant mechanism for this. In addition, they examined the effects of combining AQX-435 with the Bruton’s tyrosine kinase (BTK) inhibitor ibrutinib. Ibrutinib is also known for its inhibition of the anti-IgM-induced AKT phosphorylation, but either agent alone was only in part able to inhibit this pathway. However, in this study, it was shown that the combination of both pharmacological agents was able to achieve almost complete inhibition. The dose-dependent inhibition of the anti-IgM-induced AKT phosphorylation by AQX-435 was observed in CLL cell lines and in diffuse large B cell lymphoma (DLBCL) cells, both of which are characterized by active PI3K signaling [39].

### 4.5. SHIP1 Hyper-Activation in CLL

Ecker et al. investigated the hyper-activation of the PI3K/AKT signaling pathway and its potential role in the induction of apoptosis in 2021. They further looked at the role of SHIP1 in this context and whether negative SHIP1 modulation could be a potential therapeutic strategy. They stated that downregulation as well as hyper-activation of signaling components downstream of the B cell receptor (BCR) would lead to cell death, either through positive or negative selection. The PI3K/AKT pathway plays a pivotal role in BCR-induced intracellular signaling. First, the work group examined the effects of having a constitutively active variant of AKT in CLL cells. The AKT variant was marked with the green fluorescent protein (GFP) and transduced in CLL cells. They cultivated the transduced cells as well as the non-transduced cells and found a gradual decrease in GFP-expressing CLL cells, indicating a reduction in their viability caused by heightened AKT signaling. In view of the fact that there are currently no direct PI3K activators available, other points of intervention were determined to see if the activation of the AKT pathway may be useful in a therapeutic context. A reduction in the dephosphorylation of PtdIns(3,4,5)P_3_ represents an indirect way of activating AKT. Therefore, the authors tested the effect of the inhibition of SHIP1 by using the formerly mentioned SHIP1 inhibitor 3AC, which usually reduces the PtdIns(3,4,5)P_3_ content in CLL cells by converting it into PtdIns(3,4)P_2_. They discovered that 3AC significantly increases the activity of AKT, which subsequently leads to cell death. In addition, they were also able to show that this induction of apoptosis through SHIP1 inhibition is specific for CLL cells, whereas normal B cells did not demonstrate the same cell death-inducing effect after SHIP1 inhibition by 3AC. The authors then transferred the results into a mouse model. The CLL mouse model was treated with 3AC, and the subjects were examined regarding peripheral CLL cell infiltration. It was found that the 3AC-treated mice displayed less infiltration, and no indicators for potential toxicity could be found. This led the authors to conclude that 3AC could be a potential pharmaceutical agent for in vivo use. Next, they looked into the possible cellular mechanism for the previously observed induction of apoptosis in SHIP1-suppressed CLL cells. It is proposed that cell death is mediated by reactive oxygen species (ROS) like hydrogen peroxide and superoxide anions. Upon the inhibition of SHIP1 in MEG-1 cells and CLL cells with 3AC, the work group found a raised oxygen consumption rate in comparison to normal B cells. They state that electrons escape the oxidative phosphorylation cascade in the inner mitochondrial membrane and are transferred to oxygen prematurely, which leads to the formation of those ROS. The fact that the treatment with a mitochondria-targeting antioxidant reduced overall intracellular ROS levels seems to prove that the ROS are derived from the oxidative phosphorylation in the mitochondria. Furthermore, they found that SHIP1 inhibition in CLL cells leads to the translocation of calreticulin to the surface of the plasma membrane as well as to the excretion of danger- or damage-associated molecular patterns (DAMPs), both of which induce cell death via phagocytosis [40].

### 4.6. AQX-1125 as a Small Molecule Activator of SHIP1

AQX-1125 is a cyclohexanol with the molecular formula C_22_H_39_NO_4_, which functions as a small molecule activator of SHIP1 and was shown to directly bind to the C2 domain-containing SHIP1 protein [45]. In vitro, the k_cat_/K_M_ ratio compared with the non-pharmacologically influenced catalytic activity was reported to be increased by a factor of approximately 1.118 [45]. The authors attribute the effect to a possible change in the conformation of the protein, resulting in enhanced substrate binding and catalytic efficiency [45].

AQX-1125 has been tested in three phase 2 studies: one randomized controlled trial (RCT) for the treatment of atopic dermatitis [46], one for the treatment of pain in patients with interstitial cystitis [47], and one study of an anti-inflammatory drug in patients with exacerbations of their chronic obstructive pulmonary disease (COPD) [48]. All of the above have been completed. Also, a phase 3 RCT for the treatment of interstitial cystitis with AOX-1125 has been completed [49]. The phase 2 study on interstitial cystitis revealed that oral treatment with AQX-1125 reduces pain scores in women with moderate to severe bladder pain levels [41]. Other publications regarding those trials are not yet available.

## 5. The Role of SHIP2 in Human Diseases

**Figure 5:**
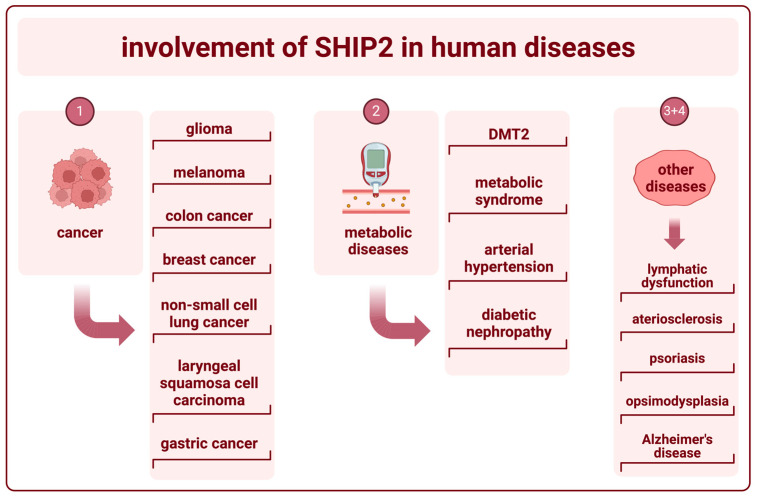
Involvement of SHIP2 in human diseases (created with BioRender). The numbers herein refer to the paragraphs with the corresponding subheading numbers under “5. The role of SHIP2 in human diseases”, wherein the involvement of SHIP2 in the pathogenesis of those diseases is further elucidated.

Polymorphisms and changes in the expression of SHIP2 are associated with several diseases in humans. The research on SHIP2 encoded within the *INPPL1* gene has been focused on its potential as an oncogene or tumor suppressor. However, SHIP2 appears to also play a major role in the pathogenesis of metabolic diseases as well as neurodegenerative diseases (Figure 5).

### 5.1. SHIP2 in Cancer

In contrast to SHIP1, SHIP2 is expressed ubiquitously and therefore has been implicated as being involved in the emergence and progression of several different types of cancers. The overexpression of SHIP2 has been reported in glioma, melanoma, colon cancer, and breast cancer and is shown to be associated with poor prognosis [1]. It is proposed by Pedicone et al. that increased cell migration and invadopodia maturation are possible mechanisms for the more aggressive nature of those epithelial cell-derived cancers. Specifically in glioma, SHIP2 inhibition was reported to reduce cell migration, which supports this hypothesis. In contrast, the authors mention that higher expression of miR-205, which targets the *INPPL1* gene encoding for SHIP2, has been associated with unknown alterations in melanocytes, which ultimately lead to their transformation into melanoma cells [1]. Furthermore, intracellular localization seems to influence important cell functions [1]. SHIP2 is predominantly localized in the nucleus and paranuclear region and is reported to be recruited to the cytoplasm, the plasma membrane, and into nuclear speckles when phosphorylated at Ser132 [50]. It is believed that SHIP2 can alter the phosphatidylinositol (PtdIns) composition in the nuclear membrane analogously to in the plasma membranes, which may lead to chromatin remodeling and the regulation of gene transcription as well as altered pre-mRNA splicing [1]. Lastly, Pedicone et al. point out that SHIP2 could simply induce resistance to cell death by increasing AKT signaling, as does SHIP1. One more study looked at SHIP2 expression in colorectal cancer and evaluated its correlation with the disease itself and different markers of disease severity. They discovered that the transcription of *INPPL1* into mRNA as well as protein expression was correlated with increased lymph node metastasis, more distant metastasis, and reduced overall survival in patients with colorectal cancer [51]. Further, SHIP2 expression was found to be elevated in colorectal cancer tissue in contrast to in non-cancerous tissue [51]. Another study looked at PtdIns(3,4,5)P_3_ phosphatases and their role in the pathogenesis of breast cancer. They discovered a correlation between increased expression of *INPPL1* and estrogen receptor negative (ER-negative) primary breast cancer, as well as grade of invasiveness and reduced survival [52]. The authors suggest that SHIP2 could promote the formation of breast tumors through activation of the AKT signaling as well as through regulation of the turnover of epidermal growth factor receptors (EGFRs). SHIP2 is believed to interact with an E3 ubiquitin-protein ligase, encoded by the *CBL* gene, thereby undermining its ability to ubiquitinate the EGFR. Seeing ubiquitination as the main driver of the internalization of proteins into intracellular lysosomes, the EGFR is more infrequently broken down, and EGF signaling is increased. However, SHIP2 knockout in mice has not been reported to trigger the formation of breast cancer [52]. Another study examined the difference in SHIP2 expression in non-small cell lung cancer (NSCLC) cells in comparison to non-cancerous cells. They found that both mRNA transcription and protein expression were heightened in NSCLC cells [22]. Furthermore, they found a positive correlation between SHIP2 protein expression and lymph node metastasis, tumor grade, and reduced 5-year survival rate [22]. Other research examined the connection between SHIP2 expression and the occurrence of laryngeal squamosa cell carcinoma (LSCC) as well as parameters of severity. The researchers came across a correlation between SHIP2 levels and the occurrence of cell degeneration, as well as between expression and tumor stage, metastasis, recurrence, and shortened disease-free survival and overall survival, in 42 LSCC patient samples in comparison to normal samples [53]. One study mentioned the potential role of SHIP2 in the pathophysiological mechanisms of cagA-positive *Helicobacter pylori* infections [54]. Those infections, if chronic, are said to be the strongest risk factor for the development of gastric cancer [55]. The bacteria transfer the cagA-protein by bacterial type IV secretion into gastric epithelial cells [55]. The formerly mentioned study suggested that SHIP2 interacts with the cagA-protein in a way that strengthens the attachment of *Helicobacter pylori* to the target cell [54].

### 5.2. SHIP2 in Metabolic Diseases

The authors of one study suggest that SHIP2 polymorphisms may take part in the pathogenesis of several diseases, which are primarily caused by disturbances in glucose and insulin homeostasis, like diabetes mellitus type 2 (DMT2) and metabolic syndrome as well as hypertension [19]. They cited another paper in which the authors suggested that a deletion in the 3′-UTR of SHIP2 was found in individuals with DMT2 more often than in healthy individuals [56]. This deletion is suggested to possibly cause an increased expression of SHIP2 and subsequently insulin insensitivity [56]. It was shown that SHIP2 upregulation inhibits insulin-stimulated AKT activation and therefore lowers the rate of glucose transporter 4 (GLUT4) translocation to the plasma membrane, which leads to reduced glucose uptake [19]. Another cross-sectional study with a high number of participants (n = 1074) investigated the *INPPL1* gene with respect to a possible correlation with the metabolic syndrome and diabetic nephropathy in Finnish people with diabetes mellitus type 1 (DMT1) [57]. They could identify two single-nucleotide polymorphisms that seem to be correlated with the existence of the metabolic syndrome in men but not in women. They could not find an association between *INPPL1* gene variants and diabetic nephropathy [57].

### 5.3. The Role of SHIP2 in Vascular Function

SHIP2 also seems to be relevant in the function of the blood and lymph vasculature. One study examined the role SHIP2 plays in the function of the lymphatic vasculature. They found one rare mutation in the *INPPL1* gene, which is associated with lymphatic dysfunction [58]. This T180A loss-of-function mutation resulted in decreased migration, tube formation, and chemotaxis to the hepatocyte growth factor (HGF) as well as decreased adhesion to collagen when overexpressed in lymphatic endothelial cells [58]. Another study by DeKroon et al. aimed to establish an association between SHIP2 and arteriosclerosis, as well as the causal pathway. They state that the anti-apoptotic function of HDL in endothelial cells is inhibited by VLDL loaded with the apolipoprotein E4 (ApoE4). They demonstrated that VLDL-ApoE4 reduced PtdIns(3,4,5)P_3_ content in the plasma membrane of endothelial cells through the activation of SHIP2, which subsequently inhibited the HDL-induced AKT signaling [59].

### 5.4. SHIP2 in Other Diseases

Two additional studies investigated the functional role of SHIP2 in other diseases. In the first study by Zibert et al., they looked at miRNA interactions and their involvement in the pathogenesis of psoriasis. They compared the expression of miRNA in normal tissue and in skin with psoriasis involvement. They found 42 miRNAs to be upregulated, of which miR-205 was one. miR-205 is a known regulator of the expression of SHIP2. The authors found a subsequent downregulation of SHIP2. They suggest that micro-RNA miR-205 acts anti-apoptotic through the downregulation of SHIP2 [60]. Finally, one review article mentioned mutations in the *INPPL1* gene to be causal for opsismodysplasia (OPS), a sort of skeletal dysplasia [3], which is primarily characterized by the delayed ossification of maturing bones [61]. The protein variants that have been associated with this disease are R401W, P659S, W688C, and F722I [3]. Furthermore, they mentioned the correlation between the upregulation of *INPPL1* and cognitive regression in Alzheimer’s disease [61].

## 6. SHIP2 Modulation as a Treatment in Human Diseases

Two studies have proposed specific methods for the treatment of type 2 diabetes and Alzheimer’s disease by modulation of SHIP2 (Figure 4). The authors of one study worked towards the discovery of small-molecule SHIP2 inhibitors in order to potentially propose therapeutic usage in diabetes mellitus type 2 (DMT2). They identified AS1949490, which inhibits SHIP2 selectively in comparison to other intracellular phosphatases [62]. To further investigate the function of this compound, they used mouse models and found that the acute administration of AS1949490 reduced the expression of gluconeogenic genes in their livers. Next, they used diabetic mice and found lowered plasma glucose levels and improved glucose intolerance when chronically treated with said compound. They mentioned the activation of insulin-induced intracellular signaling to be in part responsible for the previously described findings [62]. The other study demonstrated that the simultaneous inhibition of SHIP1 and SHIP2 in microglia can increase lysosomal compartment size, which leads to elevated capacity for the phagocytosis of non-viable neurons and amyloid beta peptides [63]. Therefore, they suggest that SHIP1/SHIP2 inhibitors could be used as treatment for Alzheimer’s disease (AD) and possibly other types of dementia. The requirements would be that the drug is able to pass the blood–brain barrier and that heightened microglial function is beneficial to the outcome [63].

### 6.1. AS1949490 as a SHIP2 Inhibitor in DMT2

Suwa et al. examined the function of the SHIP2 inhibitor AS1949490. They used a cell-based ELISA to determine the role of this compound as a potent activating agent of insulin-induced AKT phosphorylation and activation. Next, they examined the metabolic effects concerning glucose metabolism. They showed that treatment with AS1949490 increased glucose uptake in L6 myotubes as well as decreased insulin-dependent gluconeogenesis in cultured hepatocytes. In order to show that the effect is also triggered in vivo, healthy mice were treated with the compound. They discovered decreased expression levels of gluconeogenesis-related genes. They also found decreased blood glucose levels in diabetic mice after treatment with AS1949490 over seven or ten days while preserving insulin levels and body weight with no alteration in food intake [62].

### 6.2. Pan-SHIP1/SHIP2 Inhibitor K161 in AD

Pedicone et al. looked into the possibility of simultaneously inhibiting SHIP1 and SHIP2 with pan-SHIP1/2 inhibitors. They primarily investigated the possible effects on microglia cells. At first, they wanted to know whether a pan-SHIP1/2 inhibitor was able to promote the proliferation of microglial cells in the same way it is described for macrophages and osteoclasts in the literature. Therefore, BV-2 microglial cells and primary microglial cells were treated with 3AC, AS1949490, and three different pan-SHIP1/2 inhibitors K118, K149, and K161. The pan-SHIP1/2 inhibitors were the only ones to significantly increase the proliferation of both cell types. Next, the work group showed that K118 demonstrates a more potent effect on the expansion of the lysosomal compartment of BV-2 cells compared to 3AC, AS1949490, and a control. 3AC had no significant effect in this respect. They further tested the same in primary microglia with K161, 3AC, and AS194940 and discovered an effect only in the pan-SHIP1/2-treated cells. After this, the group wanted to determine whether the increased lysosomal compartment ultimately leads to increased phagocytosis of dead neurons. They tested this again in BV-2 and primary microglia cells. In the BV-2 cell group, K118, 3AC, and AS194940 were tested, and only the pan-SHIP1/2 inhibitor K118-treated cells showed significantly increased uptake of the dead neurons. In the primary microglia cells, all three formerly mentioned pan-SHIP1/2 inhibitors were tested. All three groups showed significantly enhanced phagocytosis of the dead neurons in comparison to the control, where K161 showed the best performance. At last, the group investigated the phagocytic potential of primary microglia regarding the Amyloid-β_1-42_ in response to simultaneous SHIP1 and SHIP2 inhibition. Here, they included K116 in addition to the formerly mentioned pan-SHIP1/2 inhibitors and found that all compounds but K116 increased the uptake of Aβ_1-42_. They then transferred the findings into a mouse model but only used the most promising compound, K161. They were able to show that treatment with K161 leads to significant enhancement of the phagocytic capability of mouse microglia regarding both dead neurons and Aβ_1-42_ [63].

## 7. Discussion and Outlook

As summarized in this review, SHIP1 and SHIP2 both play significant roles in the development of several human diseases. However, there is still a lot to be learned about the significance and the exact functional roles of the SHIP proteins in those diseases described above. Specifically, cancer research is a field that appears to be of great importance. It is well known that proteins that modify the phosphoinositide and/or the inositol phosphate content of a cell can act as tumor suppressors or as oncogenes. The reason is that the membrane-associated phosphoinositides and the soluble inositol phosphates often act as second messengers and activate intracellular signaling pathways in a variety of ways. Aside from SHIP1 and SHIP2, there are nine other inositol 5-phosphatases, which are encoded by the genes *INPP5A*, *INPP5B*, *INPP5E*, *INPP5F*, *INPP5J*, and *INPP5K*, as well as *SYNJ1*, *SYNJ2*, and *FIG4*. The fact that all of these different proteins modify inositol phosphates at the 5′ position of the inositol ring indicates the relevance of this modification in physiological processes. The complex interactions of all of those inositol 5-phosphatases as well as their individual and overlapping functions in healthy cells and their participation in the pathogenesis of human diseases have to be elucidated in the future.

## Data Availability

Not applicable.
